# The Potential of Targeting Ribosome Biogenesis in High-Grade Serous Ovarian Cancer

**DOI:** 10.3390/ijms18010210

**Published:** 2017-01-20

**Authors:** Shunfei Yan, Daniel Frank, Jinbae Son, Katherine M. Hannan, Ross D. Hannan, Keefe T. Chan, Richard B. Pearson, Elaine Sanij

**Affiliations:** 1 Research Division, Peter MacCallum Cancer Centre, Melbourne, VIC 3000, Australia; Shunfei.yan@petermac.org (S.Y.); dfrank@student.unimelb.edu.au (D.F.); jinbae.son@petermac.org (J.S.); ross.hannan@anu.edu.au (R.D.H.); keefe.chan@petermac.org (K.T.C.); 2 Sir Peter MacCallum Department of Oncology, The University of Melbourne, Parkville, VIC 3010, Australia; 3 Department of Biochemistry and Molecular Biology, University of Melbourne, Parkville, VIC 3010, Australia; kate.hannan@anu.edu.au; 4 The John Curtin School of Medical Research, The Australian National University, Canberra, ACT 2601, Australia; 5 Department of Biochemistry and Molecular Biology, Monash University, Clayton, VIC 3168, Australia; 6 School of Biomedical Sciences, University of Queensland, Brisbane, QLD 4072, Australia; 7 Department of Pathology, University of Melbourne, Parkville, VIC 3010, Australia

**Keywords:** high-grade serous carcinoma, ribosome biogenesis, Pol I, CX-5461, homologous recombination

## Abstract

Overall survival for patients with ovarian cancer (OC) has shown little improvement for decades meaning new therapeutic options are critical. OC comprises multiple histological subtypes, of which the most common and aggressive subtype is high-grade serous ovarian cancer (HGSOC). HGSOC is characterized by genomic structural variations with relatively few recurrent somatic mutations or dominantly acting oncogenes that can be targeted for the development of novel therapies. However, deregulation of pathways controlling homologous recombination (HR) and ribosome biogenesis has been observed in a high proportion of HGSOC, raising the possibility that targeting these basic cellular processes may provide improved patient outcomes. The poly (ADP-ribose) polymerase (PARP) inhibitor olaparib has been approved to treat women with defects in HR due to germline *BRCA* mutations. Recent evidence demonstrated the efficacy of targeting ribosome biogenesis with the specific inhibitor of ribosomal RNA synthesis, CX-5461 in v-myc avian myelocytomatosis viral oncogene homolog (MYC)-driven haematological and prostate cancers. CX-5461 has now progressed to a phase I clinical trial in patients with haematological malignancies and phase I/II trial in breast cancer. Here we review the currently available targeted therapies for HGSOC and discuss the potential of targeting ribosome biogenesis as a novel therapeutic approach against HGSOC.

## 1. Introduction

Ovarian cancer (OC) is the seventh most common cancer in females worldwide (8th overall), with over 239,000 new cases diagnosed every year. It is one of the most lethal gynecological cancers causing more than 152,000 deaths worldwide per year [1]. OC patients typically present with advanced disease at diagnosis due to the location of the disease and the lack of symptoms in early stages [2]. The five-year survival rate of 46.2% has not improved over the past three decades in contrast to other cancers such as breast cancer, which has improved from 74.9% to 89.7% [3]. Clearly, identification of new therapeutic strategies and methods of early detection are essential to achieve better patient outcome.

OC is a highly heterogeneous disease characterized by distinct clinical features including grade, histology, molecular alterations and response to therapy. OC is classified into three main types, namely epithelial (EOC), germ cell and stromal. EOC accounts for >90% of all OC cases with the majority appearing to originate from the distal fallopian tube [4,5]. Non-epithelial cancers of the ovary arising from the germ and stromal cell layers are rare, heterogeneous and have proved difficult to study [6].

EOC tumours are classified into two distinct groups, low-grade (type I) and high-grade (type II) tumours, with unique histological, clinical and molecular profiles [7,8,9,10,11] (Table 1). Low-grade serous (LGSOC), clear cell, endometrioid and mucinous ovarian cancers are categorized as type I since these tumours are confined to the ovary and are not invasive. While type II tumours comprise high-grade serous OC (HGSOC), high-grade endometrioid, undifferentiated carcinoma and carcinosarcomas. HGSOC is the most aggressive subtype and accounts for approximately 70% of all EOC [12], and is by far the most studied OC.

In addition to histological subtype, OC has been classified by different criteria. For example, Bowtell and colleagues grouped serous and endometrioid OC into six molecular subtypes (C1–C6) based on gene expression patterns including stromal, mesenchymal, immune, cell motility, cell surface/secreted markers, β catenin/T cell factor (TCF) /lymphoid enhancer factor (LEF) transcriptional targets and mitogen-activated protein kinase (MAPK) pathway activation signatures [13]. These molecular profiles are associated with different clinical outcomes and micro-environmental features such as immune and stromal cell activation [11]. Furthermore, Mori and colleagues identified five distinct molecular subtypes of EOC (Epi-A, Epi-B, Mes, Stem-A and Stem-B) that exhibited distinct clinical pathological characteristics and rates of overall survival. These subtypes show different enrichment in fibrinolysis, metastasis, extracellular matrix remodeling, TGFβ and chromatin modification processes and offer novel diagnostic and therapeutic strategies to target specific subtypes of EOC [14]. In the clinic, however, histological subtypes remain the conventional classification method for diagnosis and treatment.

Indeed, emerging knowledge of the underlying molecular alterations in EOC could allow for more personalized diagnostic and therapeutic strategies. *PIK3CA*, *BRAF* and *KRAS* somatic mutations are relatively common in type I tumours, with each subtype exhibiting distinct molecular profiles (Table 1). In contrast, HGSOCs display high levels of genomic instability with few common mutations, other than the tumour suppressor gene *TP53*, which is altered in over 90% of HGSOC cases [8,15]. Approximately, 50% of HGSOC is characterized by frequent genetic and epigenetic alteration of the Fanconi anemia/breast-related cancer antigens (BRCA) homologous recombination (HR) DNA repair pathway, most commonly affecting the *BRCA1* and *BRCA2* genes (15%–20%) [8,16,17,18]. Furthermore, the phosphatidylinositol-4,5-bisphosphate 3-kinase (PI3K) and retrovirus-associated DNA sequences (RAS) signalling pathways are altered in 45% of HGSOC cases due to loss or amplifications of genes involved in these signalling networks [8]. While most HGSOC have an initial favourable response to platinum-based therapy, this is followed by cycles of relapse and the development of acquired resistance to chemotherapy [11]. Thus, identification of new therapeutic strategies is essential to better treat this disease at diagnosis. Undoubtedly, more tailored treatments based on the molecular characteristics of the OC subtypes underpin the next phase of personalized medicine in OC.

## 2. Current Diagnostic and Standard Therapeutic Approach for High-Grade Serous Ovarian Cancer (HGSOC)

At present, there are no proven effective screening strategies for early diagnosis of HGSOC and OC in general, although a few biomarkers have been introduced to the clinic. One of the most widely studied biomarkers for HGSOC is serum levels of cancer antigen 125 (CA-125), which has been shown to correlate with disease stage and response to treatment [19]. However, a significant proportion (~20%) of early stage OC do not express the CA-125 antigen, thus tests for this marker are often used in combination with other tests, such as transvaginal sonography [20]. Furthermore, the specificity for detecting OC alone is poor [19,21,22]. False-negative diagnostic results are frequent since HGSOC may be invasive despite small tumour volume. Thus, it is important to continue to identify and validate additional biomarkers to achieve better detection of early-stage ovarian cancer.

The standard therapeutic approach for treating HGSOC relies on debulking surgery followed by subsequent treatment with a combination of platinum-based (e.g., carboplatin and cisplatin) and taxane-based (e.g., paclitaxel) drugs. Platinum-based compounds induce DNA adducts that cause DNA damage and trigger cell death [23,24]. In contrast, paclitaxel-based compounds inhibit microtubule dynamics to block mitosis, resulting in cell death [25]. Approximately 90% of patients with stage I OC (cancer confined to ovaries) show complete response to first-line treatment [26]. Patients with *BRCA1/2* mutated cancers have better outcome following platinum-based chemotherapy than their non-*BRCA1/2* mutated counterparts as their cells are incapable of repairing DNA double-strand breaks (DSBs), leading to sensitization to DNA-damaging agents [27,28]. Unfortunately, however, ~80% of patients present with advanced disease whereby tumours have metastasized to other non-ovarian tissues, which is associated with a significantly reduced response to first-line treatment [29,30]. Moreover, cases of relapse and development of chemoresistance are frequently observed [29,30]. Thus far, mechanisms of resistance include mutations that restore the original function of defective *BRCA1/2*, loss of *BRCA1* promoter methylation, *CCNE1* amplification and alteration in molecular subtype [31,32,33,34].

Several salvage regimens exist for platinum refractory cases including Topoisomerase I inhibitors (e.g., Topotecan), Topoisomerase II inhibitors (Etoposide or Doxorubicin), microtubule inhibitors (Vinorelbine), alkylating reagents (Altretamine, Ifosfamide), anti-metabolites (Gemcitabine), estrogen receptor (ER) inhibitors (Tamoxifen, in ER-positive tumours) and epidermal growth factor receptor 2 (HER2) inhibitors (Herceptin, in HER2-positive tumours). These drugs can either be used as single agents or in combination with other drugs [35,36]. However, the response rate is very low and average survival times are extremely short [2]. Indeed in April 2016, the National Institute for Health and Care Excellence (NICE) published guidance for five OC drugs, recommending two and rejecting three. In particular, the guidance recommended paclitaxel and pegylated liposomal doxorubicin hydrochloride (PLDH) for treating recurrent OC. Paclitaxel and PLDH can be used as monotherapy, or in combination with platinum.

The rapid advance of molecular discoveries in OC has offered exciting opportunities for targeted therapies. These include drugs that target the tumour vasculature as well as those that inhibit DNA repair processes or the PI3K and RAS growth signalling pathways. One of the first promising targeted agents is bevacizumab, a recombinant humanized monoclonal IgG antibody that targets vascular endothelial growth factor (VEGF) A, which has now been approved in stage IIIb recurrent platinum sensitive and refractory OC [37,38]. Although clinical trials showed remarkable improvement in progression-free survival, this did not translate to an overall survival benefit [39,40].

The identification of synthetic lethality using the poly (ADP-ribose) polymerase (PARP) inhibi­tors (PARPi) in *BRCA1*/2-mutant ovarian cancer cell lines [41] led to the development of one of the most exciting new classes of targeted therapy in HGSOC [42]. The PARP inhibitor olaparib has been approved as monotherapy for women with germline *BRCA1/2* mutations [43]. PARP inhibitors silence the PARP1/2 proteins, which are essential for sensing genotoxic insults, such as DNA single-strand breaks (SSBs) and DSBs and are involved in a number of DNA repair pathways including base-excision repair (BER) and HR [44]. These inhibitors show efficacy in cancer cells with HR deficiency (HRD) including non-*BRCA1/2* mutant tumours that carry mutations in genes involved in the HR pathway, since the accumulation of damaged DNA eventually leads to cell death. However, mechanisms leading to HR restoration have been implicated in conferring resistance to PARPi [44]. More recently, results in trials using the PARP inhibitor niraparib showed positive benefit in OC patients regardless of germline *BRAC1/2* mutations or HRD status, suggesting that HRD status may not be an effective biomarker of sensitivity to PARP inhibitors [45].

Other compounds in clinical trials showing promise include APR-246 (also known as PRIMA-1^MET^), which reactivates mutant p53 by facilitating its refolding to wild-type conformation leading to induction of apoptosis in cancer cells [46,47]. Furthermore, recent findings demonstrated the ability of APR-246 to resensitize p53-mutant OC to cisplatin, suggesting significant synergy, which may dramatically improve the outcome of HGSOC [48]. At this stage, the Phase Ib/II study of APR-246 in combination with platinum-based therapy in OC is ongoing.

Other targeted therapies in OC including targeting the oncogenic PI3K/mammalian target of rapamycin complex 1 (mTORC1) and RAS pathways remain less effective due to the complexity and redundancy of these pathways [49].

## 3. Deregulation of Growth Signalling Pathways Upstream of Ribosome Biogenesis in HGSOC

**Figure 1:**
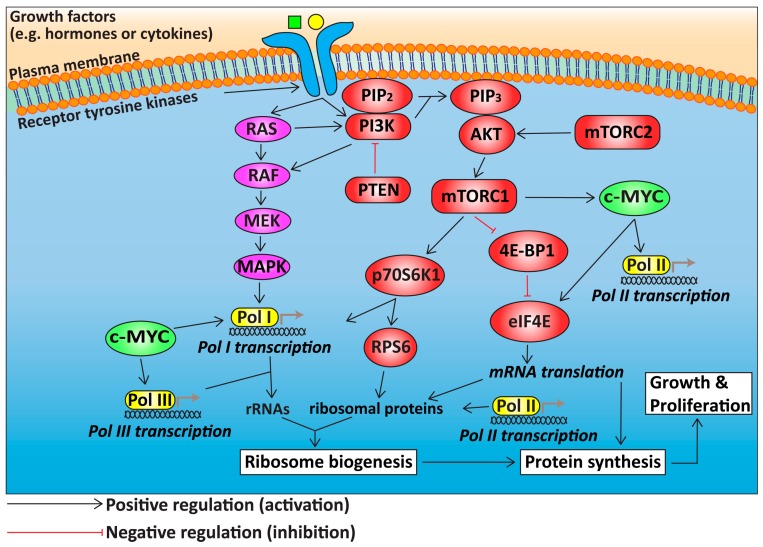
The phosphatidylinositol-3-kinase/mammalian target of rapamycin (PI3K/mTOR), retrovirus-associated DNA sequences/mitogen activated protein kinase (RAS/MAPK) pathways and c-MYC are master regulators of cell growth and proliferation. The PI3K/mTOR and RAS/MAPK signalling, and c-MYC transcription pathways are three major oncogenic drivers of cell growth and proliferation. They form a “super” network to regulate ribosome biogenesis and protein translation. The signalling cascades are predominantly initiated upon growth factor stimulation through receptor tyrosine kinases. Growth factors are represented by the green square and yellow circle. Activation of PI3K leads to induction of downstream effects mediated by the AKT oncoprotein including the activation of mTORC1 and subsequent phosphorylation of its downstream effectors, 4E-BP1 and p70S6K1. Concomitantly, the RAS signalling pathway and the c-MYC transcription factor contribute to the control of ribosome biogenesis via modulating the synthesis of rRNAs by Pol I. Moreover, c-MYC also exerts its positive regulatory effects on Pol II, Pol III, and eIF4E. Together, these three key pathways promote ribosome synthesis and protein synthesis and thus cell growth. (PI3K: phosphatidylinositol-3-kinase; mTORC: mammalian target of rapamycin complex; PTEN: phosphatase and tensin homolog; PIP2: phosphatidylinositol-4,5-bisphosphate; PIP3: phosphatidylinositol-3,4,5-triphosphate; p70S6K1: p70 S6 kinase 1; 4E-BP1: eukaryotic initiation factor 4E-binding protein 1; eIF4E: eukaryotic translation initiation factor 4E; rRNA: ribosomal RNA; Pol I: RNA Polymerase I; Pol II: RNA Polymerase II; Pol III: RNA Polymerase III). The grey arrows denote ongoing transcription.

Several signalling pathways central to normal control of cell growth and proliferation are frequently dysregulated in OC. In particular, activation of the PI3K/AKT/mTORC1 and RAS/MAPK signalling pathways and the c-MYC (v-myc avian myelocytomatosis viral oncogene homolog) proto-oncogene “super” growth regulatory network is prevalent among the major histological subtypes (Figure 1). It is now well accepted that therapeutic targeting of the PI3K/mTOR pathway alone is not sufficient for robust clinical responses in many tumour types, due to feedback loops and compensatory activation of RAS signalling [49]. Indeed, recent in vitro and in vivo pre-clinical studies demonstrate efficacy with PF502 and PD901 inhibitors, which target the PI3K/mTOR and RAS/MAPK networks respectively, to achieve significant reduction in tumour burden and improvement in overall survival [50,51]. Hence, combinatorial targeting of multiple growth signalling pathways and/or processes required for cell growth and proliferation downstream of these pathways such as ribosome biogenesis may prove effective in OC treatment. Ultimately, these pathways converge to regulate ribosome biogenesis and protein synthesis. Over the last five years, targeting ribosome biogenesis has emerged as a novel approach for cancer therapy. In this section, we review the signalling pathways upstream of ribosome biogenesis that are aberrantly regulated in OC with a primary focus on HGSOC.

### 3.1. The PI3K/AKT/mTOR Signalling Pathway in HGSOC

Constitutive activation of the PI3K/AKT/mTORC1 pathway in OC occurs through activating mutations of PI3K-related genes, amplification of AKT signalling or inactivating mutations of phosphatase and tensin homolog deleted on chromosome TEN (PTEN) [8]. This signalling cascade controls many of the processes that are important for cancer development, e.g., the cell cycle, cell survival, metabolism, motility, angiogenesis, chemoresistance, and genomic instability [52].

Mutations of genes in the PI3K/AKT/mTOR pathway are rare in HGSOC. Instead, HGSOC is associated with frequent oncogenic copy number amplifications in *PIK3CA* (23%), *RICTOR* (6%), *RAPTOR* (4%), *AKT1/2/3* (15%) and oncogenic loss of *PTEN* in 7% of cases. However, assessment of PI3K pathway activation by immunohistochemical analyses of phosphorylated/ activated AKT and mTOR identified pathway activation in approximately 50% of HGSOC [53].

In contrast to HGSOC, aberrations in the PI3K pathway are more prevalent in the rare subtypes of OC. A total of 20% of endometrioid and 35% of clear cell OC have *PIK3CA* gene mutations, whereas PTEN loss-of-function mutations occur in 20% of endometrioid OC [8,49,54,55]. Activation of the PI3K pathway in low-grade serous ovarian cancer (LGSOC) occurs through expression of insulin-like growth factor receptor (IGF-R) [56]. Single-agent studies of PI3K pathway-directed agents targeting only one node along the pathway have demonstrated limited success in OC [57]. This is due, at least in part, to feedback loops such as compensatory activation of AKT observed after mTORC1 inhibition and crosstalk between signalling pathways including the RAS/MAPK pathway [58].

### 3.2. RAS/RAF/MEK/MAPK Signalling in HGSOC

The RAS pathway plays a critical role in the regulation of cell survival, proliferation and motility. Strikingly, gene mutations in the RAS pathway components, including *KRAS*, *BRAF*, *MEK* or *ERBB2* (also known as *HER2*), encoding the upstream regulators of MAPK, are observed in more than 68% of LGSOC [9]. In HGSOC, activation of this pathway occurs through oncogenic amplification of *MAPK* (25% of cases), *KRAS* (11%), *ERBB2* (3%), and *ERBB3* (4%) [8]. Endometrioid and mucinous OC also have a high prevalence of *KRAS* mutations, up to 30% [59] and 60% of cases, respectively. *ERBB2* amplification also occurs in a small proportion of mucinous OC cases [60]. Clinically, the MAPK/ERK kinase (MEK) inhibitor selumetinib has been explored in LGSOC with promising activity and may offer an advantage over or in combination with chemotherapy [61,62]. On the other hand, a phase II trial of selumetinib in recurrent endometrioid carcinoma achieved modest results [63]. In light of potential resistance to MEK being mediated via the PI3K/AKT pathway, early data from two studies investigating the combination of PI3K and MEK inhibitors in selected OC patients based on genomic alterations in the PI3K and RAS signalling pathway support further investigation of this combination with close monitoring of cumulative toxicities [49,64].

### 3.3. c-MYC in HGSOC

The c-MYC transcription factor plays an essential role in regulating many cellular processes including cell growth, cell-cycle progression, differentiation, and apoptosis. Frequent focal amplification of chromosome 8q24 which encodes eight genes, one of which is *c-MYC*, occurs in up to 80% of HGSOC [8]. In non-transformed cells, MYC expression is typically low and tightly regulated. Whilst mutations in the *MYC* gene have been identified, the critical determinant of its oncogenic potential is its overexpression, which contributes to global increases in gene expression [65]. Furthermore, c-MYC expression levels have been associated with chemoresistance [66].

Central to MYC’s ability to drive cell growth and proliferation is its role in promoting ribosome synthesis [67,68,69]. c-MYC controls ribosome biogenesis at multiple levels by coordinating the activity of all three RNA polymerases to produce the major constituents of ribosome particles (described in more details in Section 4). c-MYC facilitates the recruitment of RNA polymerase I (Pol I) to ribosomal DNA (rDNA) promoters, and promotes the synthesis of 47S ribosomal RNA (rRNA) precursor, which is processed to form the 18S, 5.8S, and 28S mature rRNAs [70,71,72]. In addition, c-MYC enhances Pol I transcription by increasing the pool of available core transcription factors, including the upstream binding factor (UBF), selectivity factor 1 (SL-1) and RRN3 [73]. Furthermore, c-MYC promotes Pol II transcription of mRNAs encoding ribosomal proteins (RPs) and Pol III-mediated transcription of the 5S rRNA [69], as well as translation initiation by eukaryotic translation initiation factor 4E (eIF4E) [74]. Thus, c-MYC is a master regulator of ribosome biogenesis and protein synthesis [75].

Furthermore, the PI3K/mTOR and RAS/MAPK signalling cascades cooperate with the c-MYC transcription network to enhance ribosome biogenesis and protein translation (Figure 1) [76]. Upon activation by mitogenic signals, PI3K/AKT/mTORC1 signalling modulates the translation capacity and efficiency of ribosomes and induce transient upregulation of protein synthesis [76,77,78,79,80,81]. The RAS signalling cascade cooperates with c-MYC via enhancing the activity of the Pol I core transcription factors through phosphorylation by multiple kinases including MAPK, RSK and JNK [82,83]. Given that this “super” network of PI3K, RAS and MYC nodes, that controls ribosome biogenesis, is frequently deregulated in OC, targeting ribosome biogenesis may prove effective in OC treatment and can possibly overcome resistance mechanisms that allow compensatory activation of various steps in this network.

## 4. Targeting Ribosome Biogenesis Is a Novel Approach for Cancer Treatment

**Figure 2:**
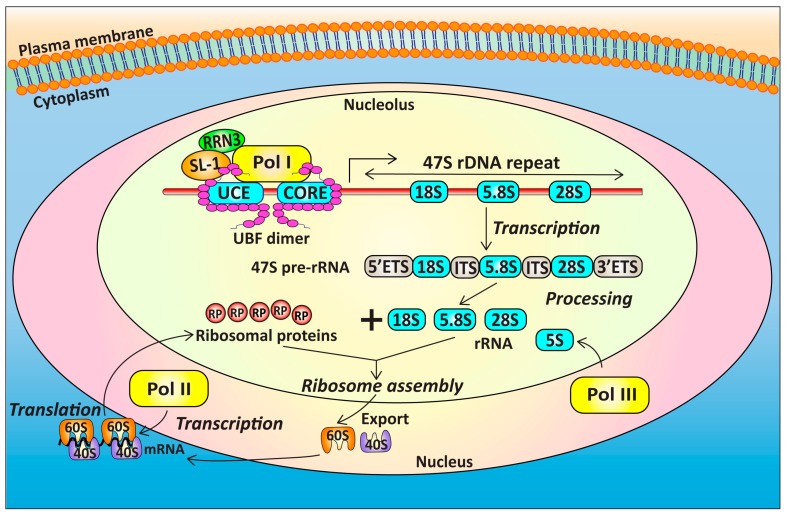
Ribosome biogenesis requires the concerted actions of Pol I, Pol II, and Pol III. The mature ribosome subunits consist of RPs and rRNAs. The Pol I-related transcription factors UBF and SL-1 interact with the rDNA promoter at the UCE and Core elements and form the preinitiation complex. Transcriptionally competent Pol I complex, defined by the presence of RRN3, is then recruited to transcribe the 47S rRNA precursor, which is processed to produce the 18S, 5.8S, and 28S mature rRNAs. These rRNAs, together with 5S rRNA transcribed by Pol III, and the RPs transcribed by Pol II are then assembled in the nucleolus to form the 40S and 60S ribosomal subunits. Upon export from the nucleolus to the cytoplasm, the fully functional 80S ribosome is then formed. (RP: ribosomal proteins; rRNA: ribosomal RNA; Pol I: RNA Polymerase I; Pol II: RNA Polymerase II; Pol III: RNA Polymerase III; PIC: Pol I pre-initiation complex; UCE: upstream control element; CORE: core promoter element; UBF: upstream binding factor; SL-1: selectivity factor 1; ETS: external transcribed spacer, ITS: internal transcribed spacer).

Synthesis of the mature 80S eukaryotic ribosomes is a tightly regulated multistep process, involving the concerted roles of Pol I, Pol II, and Pol III [84,85] (Figure 2) and utilizing at least 80% of the metabolic energy of proliferating cells [86]. The Pol I and Pol III complexes are responsible for producing the nucleic acid backbone of the mature ribosomes. Pol I transcribes the rRNA genes to produce the 47S pre-rRNA in the nucleolus, which is then processed to yield the 18S, 5.8S and 28S mature rRNA molecules [87], whereas Pol III transcribes the 5S rRNA [88]. The remainder of the ribosome is made up of approximately 78 proteins, whose mRNAs are transcribed by Pol II and, upon translation, are transported to the nucleoli and assembled with rRNAs to form the 40S and 60S ribosomal subunits, before being exported to the cytoplasm to form functional ribosomes [84,85].

Cancer cell proliferation is supported by elevated protein synthesis mediated by increased rates of ribosome biogenesis and accelerated Pol I transcription is associated with cancer development [89,90,91,92,93,94,95,96]. As such, impairing ribosome biogenesis may serve as a therapeutic approach in treating various forms of malignancy. Indeed, many cancer therapeutic drugs have been proposed to elicit their anti-tumour activity via inhibiting ribosome biogenesis [97,98,99]. Importantly, targeting Pol I transcription is now considered a promising target for cancer therapy [100]. This therapeutic approach may prove effective against HGSOC considering that deregulation of pathways upstream of Pol I transcription is a common event in HGSOC.

### 4.1. Targeting Pol I Transcription

Until 2009, only Dactinomycin (also called Actinomycin D), a naturally occurring polypeptide antibiotic that intercalates GC-rich regions of DNA, was known to be highly selective for the rRNA genes at low concentrations (5 nM) by preventing the elongation stage of Pol I transcription. In addition, the platinum compound cisplatin and inhibitors of Topoisomerase I activity (camptothecin, Irinotecan and Topotecan) inhibit Pol I transcription with some degree of specificity (reviewed in [101,102]). However, the degree to which inhibition of Pol I transcription contributes to their therapeutic efficacy is not established [99]. BMH-21, a DNA intercalator with binding preference to GC sequences, has demonstrated potent effects on rDNA transcription. BMH-1 can also target RPA194 (a core Pol I holoenzyme subunit) for proteasomal degradation leading to disassembly of the Pol I complex and dissociation from the rDNA, although this event is secondary to Pol I transcription inhibition [103]. BMH-21 achieved potent reduction of tumour growth in human melanoma and colorectal carcinoma xenograft models in vivo. Furthermore, the specific small molecule inhibitor of Pol I transcription CX-5461 has proved impressive as a novel anti-cancer agent in heamatological and prostate cancers [95,96,104,105,106]. Indeed, CX-5461 is the first specific inhibitor of Pol I transcription to enter the clinic, having progressed into “first in class” phase I clinical trials in patients with advanced haematological malignancies (Peter Mac, Melbourne, Australia) and phase I/II in breast cancer (Vancouver, BC, Canada).

### 4.2. Cellular Response to CX-5461

**Figure 3:**
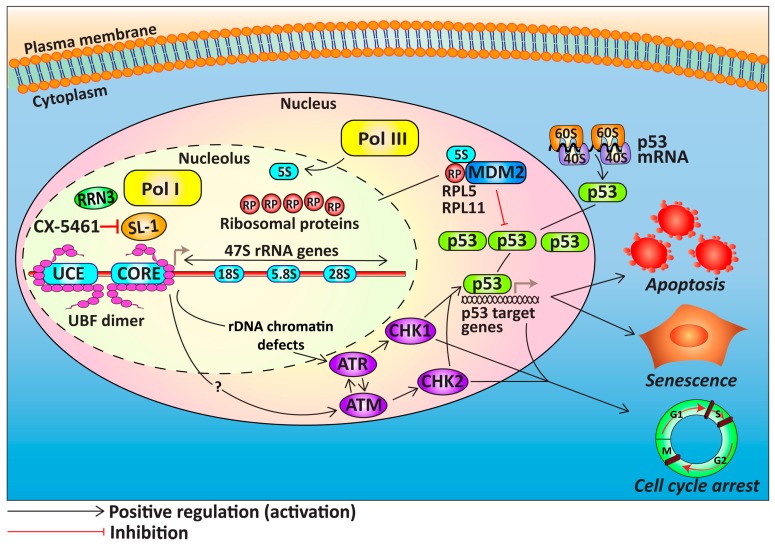
p53-dependent and -independent-mediated cellular response to CX-5461. The nucleolar stress response is initiated when ribosome biogenesis is perturbed by the Pol I transcription inhibitor, CX-5461. Central to this response is the stabilization and activation of the tumour suppressor protein p53, which triggers cell-cycle arrest, apoptosis, or senescence in a context-dependent manner. Upon alterations in ribosome biogenesis rate, free ribosomal proteins (RPs), in particular RPL5 and RPL11 in a complex with 5S rRNA, are released from the nucleolus into the nucleus to sequester the E3 ubiquitin ligase MDM2 leading to p53 stabilization. Importantly for HGSOC, CX-5461 also induces chromatin defects at rRNA genes leading to non-canonical ATM/ATR signalling and p53-independent cell-cycle arrest. (RP: ribosomal proteins; Pol I: RNA Polymerase I; UCE: upstream control element; CORE: core promoter element; UBF: upstream binding factor; SL-1: selectivity factor 1; MDM2: Mouse double minute 2 homolog; ATM: ataxia telangiectasia mutated; ATR: ataxia telangiectasia and Rad3-related protein). Grey arrows denote ongoing transcription.

CX-5461 inhibits Pol I transcription by preventing SL-1 from interacting with the rDNA promoter resulting in inhibition of Pol I recruitment and transcription initiation [104] (Figure 3). CX-5461 induces p53-dependent and independent anti-proliferative responses including cell-cycle arrest, apoptosis, or senescence in various cancer cell lines [104,107]. This includes activation of the nucleolar stress response, which is a surveillance mechanism that coordinates cellular response to deregulation of ribosome biogenesis [101,104,108]. Central to this response is the activation of p53 via the release of free RPs from the nucleolus, in particular the RPL5/RPL11/5S rRNA complex [109,110], which binds and inactivates the E3 ubiquitin ligase murine double minute 2 (MDM2) [111], thus preventing proteasomal degradation of p53 [112]. The outcomes of p53 activation are diverse, ranging from DNA repair, transient cell-cycle arrest, apoptosis, permanent cell-cycle arrest or senescence [113,114] (Figure 3). CX-5461 selectively induces p53-mediated apoptosis of MYC-driven B-lymphoma cells in vivo with minimal effects on wild type cells of the same lineage [96,104,105]. The dependence of MYC-driven B-cell lymphoma on high rates of Pol I transcription sensitizes these cells to CX-5461 while normal cells can tolerate reductions in rRNA synthesis without induction of cell death [96,104]. A recent study demonstrated that cells with a high rate of ribosome biogenesis exhibit high levels of p53 protein stabilization upon treatment with CX-5461, due to more RPs being available to bind MDM2 thus preventing p53 degradation [115].

Furthermore, studies in acute lymphoblastic leukemia (ALL) suggested that CX-5461 treatment of p53-mutant ALL cells leads to G2 phase arrest and induction of apoptosis via the ataxia telangiectasia mutated (ATM)/ataxia telangiectasia and Rad3-related protein (ATR) pathways [116,117] (Figure 3). More recently, CX-5461 was shown to induce p53-independent, ATM/ATR-mediated G1 and G2 cell-cycle arrest, and senescence in telomerase reverse transcriptase (TERT)-immortalized human fibroblasts [107]. Acute treatment with CX-5461 (1 h) was shown to induce chromatin defects at the rDNA associated with depletion of Pol I binding across the transcribed region, leading to activation of DNA damage signalling in the absence of global DNA damage [96]. Since the therapeutic efficacy of CX-5461 is not restricted to cellular p53 status, CX-5461 has the potential for treating HGSOC either as a single agent or in combination therapies.

## 5. The Potential of Targeting Ribosome Biogenesis in HGSOC

Recent studies utilizing CX-5461 in combination therapy have demonstrated impressive results in targeting the intrinsic reliance of cancer cells on ribosome biogenesis. Indeed, targeting ribosome biogenesis and protein translation by combining CX-5461 with the mTORC1 inhibitor, everolimus, synergistically reduced tumour burden and provided remarkable improvement in survival rate of MYC-driven lymphoma-bearing mice [105]. The synergistic efficacy of this combination in vivo was due to more robust suppression of Pol I transcription compared to single-agent treatment as well as the induction of tumour cell death via independent pathways. CX-5461 induced nucleolar stress and p53 pathway activation, whereas everolimus induced expression of the pro-apoptotic protein BH-only protein BCL modifying factor (BMF) [105]. While the therapeutic efficacy of CX-5461 against MYC-driven B-cell lymphoma is linked to p53-mediated apoptosis, it is not restricted to p53 status in solid cancer cell lines [104]. Everolimus is currently being evaluated in phase I trial for OC [118,119] and the combination with CX-5461 may provide benefit for the treatment of this disease. Furthermore, a recent study has provided preclinical evidence demonstrating improved therapeutic efficacy in preclinical models of MYC-driven prostate cancer upon combined treatment of CX-5461 and an inhibitor of PIM kinase [106]. The oncogenic PIM kinase promotes c-MYC transcriptional activity and stability, as well as stimulating eIF4E-dependent mRNA translation. Strikingly, CX-5461 in combination therapy with the pan-PIM kinase inhibitor CX-6258, led to a reversion of Hi-MYC tumours back to high-grade intraepithelial neoplasia. In addition, this combination showed significant efficacy in an aggressive chemotherapy-refractory high MYC patient-derived xenograft (PDX) model of prostate cancer. These findings provide a further rationale for translating this combination therapy to HGSOC.

Moreover, as described in Section 4.2, CX-5461 induces a p53-independent ATM/ATR-mediated G2 cell-cycle checkpoint. The combination of CX-5461 with a drug targeting ATM/ATR signalling led to enhanced therapeutic benefit in treating p53-null MYC-driven lymphoma in vivo, which are normally refractory to either drug alone [107]. Inhibition of DNA damage response (DDR) signalling has become an attractive therapeutic strategy in cancer treatment with highly selective small molecule inhibitors of ATM and ATR signalling in preclinical and clinical development, respectively in OC thus providing a rationale for CX-5461 combination with DDR inhibitors in HGSOC treatment. Furthermore, CX-5461 was shown to sensitize primary fibroblasts to DNA-damaging agents [107], raising the possibility of enhancing the therapeutic efficacy against HGSOC by combining CX-5461 with standard chemotherapies (carboplatin/cisplatin) or emerging targeted therapies, such as PARP inhibitors. Potentially, the combination of APR-246 with CX-5461, which could restore p53 activity and allow increased p53 protein stabilization through CX-5461-mediated nucleolar stress response, may also prove effective against HGSOC.

## 6. Conclusions

While poly ADP-ribose polymerase (PARP) inhibitors have demonstrated promising therapeutic benefits in high-grade serous ovarian cancer (HGSOC) patients [120], there are concerns regarding the complexity of mechanisms underlying resistance to PARP inhibition. Thus, the possibility of enhancing the therapeutic efficacy against HGSOC by combining CX-5461 with PARP inhibitors or standard chemotherapies is an exciting novel approach for the treatment of this disease. In addition, combined targeting of ribosome biogenesis and protein translation may also be effective, such as combining CX-5461 with mammalian target of rapamycin (mTOR) inhibitors, by overcoming the redundancy of the retrovirus-associated DNA sequences (RAS) and phosphatidylinositol-3-kinase (PI3K) signalling networks and the complexity of resistance mechanisms to pathway inhibitors. The therapeutic inhibition of these drivers has the potential to overcome genetic heterogeneity and improve patient outcome.

While this review has focused on HGSOC, there is also a strong rationale for potential trials in the other histological subtypes. Rare OC subtypes have a very different molecular profile. Given frequent and occasional co-existing alterations in the RAS and PI3K pathways in low-grade serous ovarian cancer (LGSOC), targeting ribosome biogenesis may also be appropriate in this subgroup. Furthermore, LGSOC and endometrioid ovarian tumours frequently express hormone receptors; thus, combining endocrine treatment with CX-5461 in these subtypes is worthy of investigation.

To date, CX-5461 is well tolerated with low-grade manageable adverse events in 13 patients with advanced haematological malignancies while the trial continues with dose escalation [121]. Certainly, preclinical studies utilizing CX-5461 indicate that targeting ribosome biogenesis will be most efficacious in tumours with dysregulated growth control downstream of the v-myc avian myelocytomatosis viral oncogene homolog (MYC) and PI3K nodes [96,105] and potentially deregulated DNA damage response (DDR) [107]. Understanding mechanisms of cellular response to targeting ribosome biogenesis in various tumour types, as well as identifying predictive biomarkers of response, will be crucial for the success of this novel class of targeted therapy and for the development of rational combinatorial strategies.

## Figures and Tables

**Table 1:** Histotypes and genetic alterations of OC.

Epithelial Ovarian Cancer					
Subtypes	High-Grade Serous Ovarian Cancer (Type II)	Low-Grade Serous Ovarian Cancer (Type I)	Clear Cell Ovarian Cancer (Type I)	Endometrioid Ovarian Cancer (Type I)	Mucinous Ovarian Cancer (Type I)
**Genome Instability**	High	Low	Low	Low	Low
**Frequent Genetic Alternations**	*TP53* mut (>90%)	*TP53* wt	*TP53* wt	*TP53* wt	*TP53* wt
	*BRCA1/2*(~15%) HR deficiency (up to 50%)	Uncommon	Uncommon	Uncommon	Uncommon
	**PI3K pathway** (*PIK3CA*, *RICTOR*, *AKT*, *RAPTOR*, *PTEN*) **RAS pathway** (*KRAS*, *MAPK*, *ERBB2*) **IGF-1R, EGFR, KIT, CNNE**	**RAS pathway** (*KRAS*, *BRAF*) **HER2**	**PI3K pathway** (*PIK3CA*) **MET**	**PI3K pathway** (*PIK3CA*, *PTEN*) **β-catenin**	**RAS pathway** (*KRAS*/*BRAF*) **HER2**
**5-year Survival Rate**	~40%	~70%	>70%	>90%	~78%

## Abbreviations

ALL	acute lymphoblastic leukemia
ATM	ataxia telangiectasia mutated
ATR	ataxia telangiectasia and rad3-related protein
BER	base-excision repair
BRCA	breast-related cancer antigens
CA-125	cancer antigen 125
DDR	DNA damage response
DSBs	double-strand breaks
eIF4E	eukaryotic translation initiation factor 4E
EOC	epithelial ovarian cancer
ER	estrogen receptor
HER2	human epidermal growth factor receptor 2
HGSOC	high-grade serous ovarian cancer
HR	homologous recombination DNA repair
HRD	homologous recombination DNA repair deficiency
IGF-R	insulin-like growth factor receptor
LGSOC	low-grade serous ovarian cancer
MAPK	mitogen-activated protein kinase
MEK	MAPK/ERK kinase
mTOR	mammalian target of rapamycin
mTORC1	mammalian target of rapamycin complex 1
MDM2	murine double minute 2
MYC	v-myc avian myelocytomatosis viral oncogene homolog
OC	ovarian cancer
PARP	poly ADP-ribose polymerase
PDX	patient-derived xenograft
PI3K	phosphatidylinositol-3-kinase
Pol I	RNA polymerase I
Pol II	RNA polymerase II
Pol III	RNA polymerase III
PTEN	phosphatase and tensin homolog
RAS	retrovirus-associated DNA sequences
rDNA	ribosomal DNA
rRNA	ribosomal RNA
RPs	ribosomal proteins
SL-1	selectivity factor 1
TERT	telomerase reverse transcriptase
UBF	upstream binding factor
VEGF	vascular endothelial growth factor
